# Medical Emergency Team: How do we play when we stay? Characterization of MET actions at the scene

**DOI:** 10.1186/s13049-016-0222-7

**Published:** 2016-03-22

**Authors:** Raquel Silva, Manuel Saraiva, Teresa Cardoso, Irene C. Aragão

**Affiliations:** Unidade de Cuidados Intensivos Polivalente – Hospital de Santo António, University of Porto, Largo Prof. Abel Salazar, 4099-001 Porto, Portugal

**Keywords:** Medical Emergency Team, Rapid Response Team, Cardiac Arrest Team, Efferent limb, Activation criteria, Actions at the scene, Immediate outcome

## Abstract

**Background:**

The creation, implementation and effectiveness of a medical emergency team (MET) in every hospital is encourage and supported by international bodies of quality certification. Issues such as what is the best composition of the team or the interventions performed by the MET at the scene and the immediate outcomes of the patients after MET intervention have not yet been sufficiently explored. The purpose of the study is to characterize MET actions at the scene and the immediate patient outcome.

**Methods:**

Retrospective cohort study, at a tertiary care, university-affiliated, 600-bed hospital, in the north of Portugal, over two years.

**Results:**

There were 511 MET activations: 389 (76 %) were for inpatients. MET activation rate was 8.6/1,000 inpatients. The main criteria for activation were airway threatening in 143 (36.8 %), concern of medical staff in 121 (31.1 %) and decrease in GCS > 2 in 98 (25.2 %) patients; MET calls for cardiac arrest occurred in 68 patients (17.5 %). The median (IQR) time the team stayed at the scene was 35 (20–50) minutes. At the scene, the most frequent actions were related to airway and ventilation, namely oxygen administration in 145 (37.3 %); in circulation, fluid were administered in 158 (40.6 %); overall medication was administered in 185 (47.5 %) patients. End-of-life decisions were part of the MET actions in 94 (24.1 %) patients. At the end of MET intervention, 73 (18.7 %) patients died at the scene, 190 (60.7 %) stayed on the ward and the remaining 123 patients were transferred to an increased level of care. Crude hospital mortality rate was 4.1 % in the 3 years previously to MET implementation and 3.6 % in the following 3 years (*p* < 0.001).

**Discussion:**

During the study period, the rate of activation for medical inpatients was significantly higher than that for surgical inpatients. In our hospital, there is no 24/7 medical cover on the wards, with the exception of high-dependency and intensive care units; assuming that the number of unplanned admissions and chronic ill patients is greater in medical wards that could explain the difference found, which prompts the implementation of a 24/7 ward residence.

The team stayed on site for half an hour and during that time most of the actions were simple and nurse-driven, but in one third of all activations medical actions were taken, and in a forth (24%) end-of-life decisions made, reinforcing the inclusion of a doctor in the MET. A significant decrease in overall hospital mortality rate was observed after the implementation of the MET.

**Conclusions:**

The composition of our MET with an ICU doctor and nurse was reinforced by the need of medical actions in more than half of the situations (either clinical actions or end-of-life decisions). After MET implementation there was a significant decrease in hospital mortality. This study reinforces the benefit of implementing an ICU-MET team.

## Background

The implementation of Medical Emergency Teams (MET) seems to be associated with a reduction in hospital mortality and in-hospital cardiac arrest [1]. Therefore, their creation and implementation in every hospital is encouraged and supported by international bodies of quality certification, such as the Joint Commission [2] and the Institute for Healthcare Improvement [3].

**Fig. 1:**
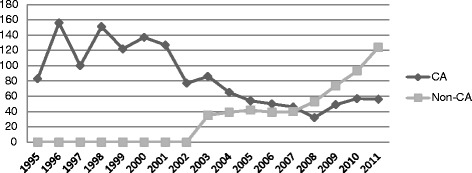
Criteria of team activation between 1995 and 2011. CA – cardiac arrest. Non-CA – No Cardiac Arrest

In 1994 the first national cardiac arrest team was organized in this hospital, by the intensive care unit, already composed by an Intensive care Unit (ICU) doctor and nurse, and it remained like that until 2010 when a MET system was implemented Between 1995 and 2010, the cardiac arrest team was activated 1811 times. Activation for non-cardiac arrest situations steadily increased, reaching 61 % of all calls in the last 3 years (2008–2010) which prompt the readjustment of the cardiac arrest team to a MET (Fig. 1).

**Fig. 2:**
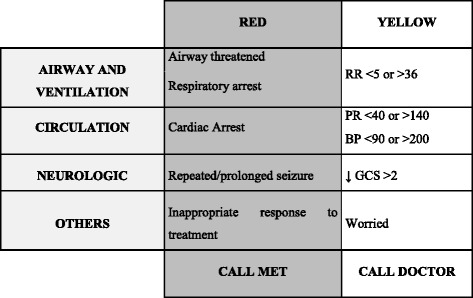
MET activation criteria. RR – respiratory rate, PR – pulse rate, BP – blood pressure, GCS – Glasgow Coma Scale

The same team is responsible for the institutional Basic Life Support (BLS) provision to all healthcare professionals. Following this evolution an emphasis on early identification of signs and symptoms of clinical deterioration that should prompt MET activation (Fig. 2) was included in the hospital Basic Life Support course.

MET is activated by a dedicated phone line in ICUand responds to calls from everywhere in the hospital on a 24/7 basis. Specific resuscitation trolleys that include equipment for resuscitation (including endotracheal intubation material) and drugs are available on all wards and specific locations around the hospital. The nurse team in each location is responsible for the maintenance of the resuscitation trolleys and the MET is responsible for auditing its maintenance.

After activation, the MET performance on site includes patient assessment and emergency treatment, referral to the most appropriate place for continuation of care and in some cases discussions about goals of clinical care which might include end-of-life decisions. Issues such as determining the best composition of the team or the ideal interventions performed by the MET at the scene as well as the immediate outcome for patients after MET intervention have not yet been sufficiently explored [4].

For this reason, the aim of this paper is to characterize MET activations and actions at the scene and the immediate patient outcome.

## Methods

Retrospective cohort study, conducted at Hospital Santo António, Centro Hospitalar do Porto, a university-affiliated, tertiary care, 600-bed hospital, in the north of Portugal.

Demographic and outcome data were obtained from the hospital administrative system. In 2012, a MET database was implemented to allow periodic audits. Data concerning team activations, with special emphasis on MET local clinical actions, was retrieved from this database.

All inpatients activations from January 2012 to December 2013 were included in the study.

The study was approved by the hospital’s institutional review board, ethics committee (Ref.143/11 – 098DEFI/124CES) and national commission for data protection.

## Results

### MET activations

During the study period (2 years), there were a total of 511 MET activations: 389 (76 %) were for inpatients and the remaining 122 (24 %) for outpatients (for instance: hospital workers, relatives or visits of inpatients, or patients that come into the outpatient clinic, pharmacy, medical exams and so on).

MET activation rate was 8.6/1,000 inpatients:11.5/1,000 medical inpatients and 6.1/1,000 surgical inpatients (*p* < 0.001).

Most MET calls happened during the night shift (20:01–8:00) with 175 calls (46 %), followed by the morning shift (8:01–14:00) with 116 calls (30 %) and the afternoon period (14:01–20:00) with 96 calls (24 %). In two patients, time of activation was not registered. During the study period, there were 506 weekdays, during which 285 MET activations occurred, resulting in 56,3 activations/100 weekdays. The same period contained 225 weekend/holidays days on which 104 MET activations occurred, resulting in 46.2 activations/100 weekend/holidays days (*p* = 0.16).

Time to reach the various locations of calls, was less than 2 min, in three random measures: 1’58”, 1’36” and 1’10”.

The group of 389 inpatients had a mean (±SD) age of 72 (±10.8) years, 224 (58 %) were male and 207 (53 %) were medical. Fifty patients (14 %) had been transferred from high dependency units and six (2 %) from intensive care in the previous 24 h.

In Table 1, criteria for MET activation are shown: “airway threatened” was the most common criterion followed by “staff worried”;, “cardiac arrest” came in fifth; in 55 % of cases, there was more than one criterion identified by the person who called.

**Table 1:** MET: Activation criteria, interventions and procedures, n(%)

MET activation Criteria^a^		N (%)
Airway threatened		143 (36.8)
Staff worried		121 (31.1)
↓ GCS > 2		98 (25.2)
BP < 90 or >200		85 (21.9)
Cardiac arrest		68 (17.5)
RR <5 or >36		38 (9.8)
PR <40 or >140		30 (7.7)
Respiratory arrest		28 (7.2)
Innapropriated Response to treatment		24 (6.2)
Repeated/prolonged seizure		15 (3.9)
Interventions and Procedures implemented by MET^b^		
Fluid challenge		158 (40.6)
Bag mask ventilation		145 (37.3)
IV access		29.8 (116)
Manual ventilator		78 (20.1)
ET		61 (15.7)
Airway suction		53 (13.6)
CPR		48 (12.3)
Oropharyngeal tube		36 (9.3)
Nebulization		25 (6.4)
Non-invasive ventilation		12 (3.1)
Blood transfusion		8 (2.1)
Cardioversion and pacing		8 (2.1)
Drugs	Intravenous vasopressor	53 (13.6)
	Intravenous anesthetics	47 (12.1)
	Others	46 (11.8)
	Intravenous antiarrithmics	30 (7.7)
	Intravenous diuretics	30 (7.7)
	Intravenous painkillers	29 (7.5)
	Bronchodilators	24 (6.2)
	Steroids	14 (4.4)
	Intravenous neuromuscular-blocking	9 (2.3)

^a^Could be more than one criterion in each activation

^b^All interventions during each MET response were included

*PR* pulse rate, *RR* respiratory rate, *BP* blood pressure, *GCS* – Glasgow Coma Scale, *ET* Endotracheal entubation, *IV* intravenous, *CPR* cardiopulmonar resuscitation

During activation, the median (IQR) time that the team stayed with the patient was 35 (20–50) minutes.

### MET actions: ABC approach

At the scene, the most frequent actions were related to airway and ventilation, namely oxygen administration in 145 (37.3 %) and bag and mask ventilation in 78 cases (20.1 %). For circulation, placement of additional peripheral venous access was performed in 116 (29.8 %) and fluid administration in 158 cases (40.6 %). In 185 (47.5 %) patients, medication was administered and in 136 (35 %) more than one type of medication was given (Table 1).

End-of-life decisions were also part of the MET actions, either as a clinical decision maker or supporter. Of all patients, 20 (5.1 %) had already a DNR order and/or a withhold therapy decision (*n* = 10, 2.5 %) before MET activation. An additional 94 (24.1 %) DNR orders were made during the MET approach. Of these, 42 (11 %) were decisions to withhold therapy.

### Immediate outcome

**Fig. 3:**
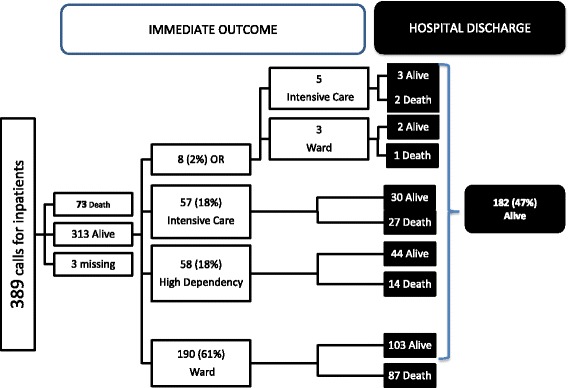
Immediate and hospital discharge outcome, n (%). OR – operating room

At the end of the MET intervention, 73 (18.7 %) patients died at the scene (Fig. 3). Of the 313 who survived, 123 (39,3 %) were transferred to the operating room, high-dependency unit (HDU) or ICU; of these, 69 cases (56.1 %) could not be transferred immediately, so they were transiently transferred to the emergency room, under the care of the same clinical team, ensuring the same level and quality of care. Two patients were transferred to another institution for cardiac surgery that is not available at our hospital.

In 190 (60.7 %) patients, there was not a need to increase the level of care, so they remained on the ward. In 36 (18.9 %) patients, this decision was related to end-of-life decisions. Of these, 33 (91.6 %) were implemented by MET.

In the group of patients alive at the end of MET intervention, 182 were alive at the time of hospital discharge (Fig. 3). In this group the hospital mortality rate was 52.8 % (*n* = 204).

Over the 2 years of the study period, 1,441 inpatients died in hospital and MET was activated for 204 (8.6 %) of these.

Among the group of patients with end-of-life decisions (*n* = 114), 23 were alive at hospital discharge (15 had a withhold therapy decision and eight a DNR order).

Patients for whom MET activation was due to cardiopulmonary arrest (*n* = 68) had a hospital mortality rate of 88 % (*n* = 60); of these 28 (41.1 %) had an end-of-life decision (5 previously and 23 during MET intervention).

Mean hospital mortality rate in the three years previously to MET implementation (2008–2010) was 4.1 % and in the following three years 3.6 % (2011–2013), *p* < 0.001).

## Discussion

The main goal of this study was to describe and analyze MET actions during activation and the immediate patient outcome.

During the study period, the MET activation rate was 8.6/1,000 inpatients, which is similar to what has been described in other studies [5, 6]. The rate of activation for medical inpatients was significantly higher than that for surgical inpatients, in previous reports some describe higher activation rates for surgical patients [7, 8] and others found no differences between both groups [9, 10]. In our hospital, there is no 24/7 medical cover on the wards, with the exception of the HDU and ICU along with the emergency department. Assuming that the number of unplanned admissions is greater in medical patients and the fact that this group has more comorbidities, it could probably explain the difference found. This finding also prompts the planning for the implementation of a 24/7 ward residence, mainly in the medical wards. As expected, we found higher activation rates on the night shift, which is the period without medical cover on the wards, surprisingly there was no difference in the activation rate between weekdays and weekends.

Fifty patients (14 %) had been transferred from HDU and six (2 %) from ICU during the previous 24 h, indicating some kind of severity of acute disease. The activation of MET in the following 24 h might reflect an abrupt downgrade of clinical care. This data also prompts the respective clinical teams to pay more attention to the presence of firm criteria of discharge.

On the other hand, the fact that the vast majority of patients who needed an upgrade in clinical care had to wait in the emergency room due to a lack of available beds indicates an insufficient number of beds for differentiated clinical care. Most of the time, the occupation rates of our HDU and ICU are above 90 %, meaning that the need for a new admission frequently prompts an early discharge. This lack of differentiated clinical care beds may not be absolute and reflect the difficulty to discharge patients to the appropriated wards.

Portugal is in the lower range of high dependency and intensive care unit (HDU-ICU) beds /100.000 population among many other countries like the UK, Ireland, Sweden, Netherlands, Greece, Finland among others (between 4.2 and 6.7/100.000) and similar to South Africa, China and New Zealand, which might explain the urge in downgrading patients. Despite that, our activation rate was similar to that reported by hospitals from countries with higher (the USA) and lower (Sweden) number of HDU-ICU beds number suggesting that our reality has external application, specially to these other countries if the structure of acute care is similar [11].

Cardiac arrest was the fifth cause for MET activation (in less than 18 % of cases), which nonetheless represents a significant number of patients. Bellomo [7, 8, 12] describes that more than 25 calls per 1,000 admissions improve MET system in terms of progressive reduction in cardiac arrest and hospital mortality; this figure is higher than our activation rate (8.6/1,000 admissions). “Staff worried” was the second most common cause for MET activation, in nearly one third of all activations, coinciding with the Bellomo [7] study.” Staff worried” should be used for any patient that does not fit any other criteria and for whom the staff are seriously concerned. We identify the need to reinforce objectivity on the criteria for MET activation, which prompts the team to make a more complete clinical assessment, including all vital signs and conscious level, in our educational program. On the other hand, in our educational program “Staff worried” should trigger a call for the attending physician and in their absence the emergency department doctor. This may cause a delay in attending to the situation and prompt the clinical team on site to activate MET. Again, this could be improved by the implementation of a 24/7 hospital residence.

The team stayed on site for half an hour, similar to what was described in the Konrad study [9], but longer than in the Bellomo study [7]. During this time, several actions were conducted, including fluid challenge, oxygen therapy, additional IV access and bag-mask ventilation. These interventions performed by the MET are technically “simple”, like described by other studies [7, 9, 13]. The focus on early recognition of clinical deterioration signs made in our educational program (BLS) might be responsible for an early activation, decreasing the complexity of care required [7], which is a satisfying result for us. Our MET response seems to be very quick (less than 2 min), quicker than what has been reported in previous studies (12.3 min in the study from Konrad [9] and 4.5 min in the study from Bellomo [7]), thus preventing further clinical deterioration. Differing from other hospitals [7, 13] our MET is always composed of an ICU doctor and nurse; this structure is reinforced by the need to perform medical actions in one third of all activations (32 %), apart from end-of-life decisions.

End-of-life decisions were made in 24 % of the patients, a higher proportion than the one described by Maharaj [1]. MET activation is seen as a sentinel event that opens the dialogue regarding goals of care that might not occur otherwise. The intervention is also important in reinforcing that withholding therapy decisions (including DNR orders) imply maintaining the same level and quality of clinical care. This was translated in the survival of 20 % of the patients that had end-of-life decisions.

More than a third of the patients who survived, stayed on the same ward. Of these, only 19 % had end-of-life decisions, reinforcing that early recognition and intervention solve an important proportion of cases, thus avoiding an upgrade of care or unplanned ICU admissions and improving patient outcome [8, 12]. Among the group of inpatients who died in hospital, only 8.6 % had a MET visit which could be a reflection that they were expected deaths for whom an upgrade in clinical care was not adequate.

A significant decrease in overall hospital mortality rate was observed after the implementation of the MET.

### Limitations

This is a single center study with a limited number of patients included in it. It was performed at a tertiary care university-affiliated hospital and therefore the findings and suggestions may not be applicable to other realities. Nevertheless, the share of facts from different clinical realities and countries is what allows the development of worldwide recommendations, and our local suggestions could be applied to similar clinical settings around the world.

Time to reach the location of the call might play a role in the outcome, but unfortunately only three random measures were taken. The target is to reach the location of the call in less than 2 min and a systematic audit is planned to determine if the team reaches the place in less than two minutes in all cases and if not what is the impact of time to scene in patient outcome.

## Conclusions

The composition of our MET with an ICU doctor and nurse was reinforced by the need of medical actions in more than half of the situations (either clinical actions or end-of-life decisions). The MET team common with the emergency room team made it easier to deal with the lack of available HDU or ICU beds while still maintaining the same level of care.

Given these results, it can be assumed that our hospital’s MET model allows a prompt attendance, assessment and adequate treatment of the deteriorating patient on the ward, thus preventing unplanned ICU admissions and improving patient outcome, with a significant decrease in hospital mortality observed after its implementation.

Reinforcement in the hospital’s educational program is needed to improve the objectivity of MET calls.

## Abbreviations

BLS: basic life support
MET: Medical Emergency Team
DNR: do not resuscitate
ICU: intensive care unit
HDU: high-dependency unit

## References

[1] Maharaj R, Raffaele I, Wendon J (2015). Rapid response systems: a systematic review and meta-analysis. Crit Care.

[2] The Joint Commission (2007). The Joint Commission 2008 National Patient Safety Goals. Jt Comm Perspect.

[3] Institute for Healthcare Improvement. 5 Million Lives Campaign: Overview. http://www.ihi.org/about/Documents/5MillionLivesCampaignCaseStatement.pdf Accessed 10 December 2012

[4] Subbe C. The Medical Emergency Team: Hospital Outcome after a Day (METHOD) study, version 2. 2013. http://rapidresponsesystems.org/METHOD/METHOD_protocol_V_15-12-2013.pdf. Accessed 20 Jan 2014.

[5] Smith R, Hayashi V, Lee Y, Navarro-Mariazeta L, Felner K (2014). The Medical Emergency Team call: a sentinel event that triggers goals of care discussion. Crit Care Med J.

[6] Campello G, Granja C, Carvalho F, Dias C, Azevedo L, Costa-Pereira A (2009). Immediate and long-term impact of medical emergency teams on cardiac arrest prevalence and mortality: a plea for periodic basic life-support training programs. Crit Care.

[7] Bellomo R, Goldsmith D, Uchino S, Buckmaster J, Hart GK, Opdam H (2003). A prospective before-and-after trial of a medical emergency team. Med J Aust.

[8] Jones D, Bellomo R, DeVita M (2009). Effectiveness of the Medical Emergency Team: the importance of dose. Crit Care.

[9] Konrad D, Jaderling G, Bell M, Granath F, Ekbom A, Martling C (2010). Reducing in-hospital cardiac arrests and hospital mortality by introducing a medical emergency team. Intensive Care Med.

[10] Moldenhauer K, Sabel A, Chu E, Mehler P (2009). Clinical triggers: an alternative to a rapid response team. Jt Comm J Qual Patient Saf.

[11] Rhodes A, Ferdinande P, Flaaten H, Guidet B, Metnitz PG, Moreno RP (2012). The variability of critical care bed numbers in Europe. Intensive Care Med.

[12] Jones D, DeVita M, Bellomo R (2011). Rapid-response Teams. N Engl J Med.

[13] Chan P, Khalid A, Longmore L, Berg R, Kosiborod M, Spertus J (2008). Hospital-wide code rates and mortality before and after implementation of a rapid response team. Am Med Assoc.

